# Supplementary material for: Genomes of the Caribbean reef-building corals Colpophyllia natans, Dendrogyra cylindrus, and Siderastrea siderea
Source: bioRxiv. 2024 Aug 22:2024.08.21.608299. Preprint. [Version 1] doi: 10.1101/2024.08.21.608299 (PMC11370458; doi:10.1101/2024.08.21.608299)
Supplement: Supplement 1 [file media-1.pdf]

Supplementary Data

Supplemental Tables

Table S1: Summary statistics of each Pacific BioSciences SMRTCell, for *Colpophyllia natans, Dendrogyra cylindrus*, and *Siderastrea siderea*. Gb=gigabases

	PacBio SMRTCell								
	1			2			3		
	C. natans	*D. cylindrus*	*S. siderea*	*C. natans*	*D. cylindrus*	*S. siderea*	*C. natans*	*D. cylindrus*	*S. siderea*
Mean read length	8,748.90	8,832.50	8,638.70	8,652.30	8,744.80	8,532.00	11,351.60	11,486.10	11,122.90
Mean read quality	44.6	42.5	43.1	45	42.9	43.6	38.7	37.2	37.9
Median read length	8,528.00	8,610.00	8,412.00	8,458.00	8,555.00	8,331.00	11,036.00	11,177.00	10,848.00
Median read quality	41.2	40	40.3	40.9	39.8	40.1	37	36.1	36.4
Number of reads	1,021,217	999,811	972,903	1,086,357	1,081,499	1,118,551	709,215	612,536	1,314,195
Read length N50	9,191.00	9,271.00	9,068.00	9,164.00	9,242.00	9,031.00	12,108.00	12,131.00	11,908.00
STDEV read length	2,359.40	2,342.60	2,340.80	2,419.80	2,401.70	2,401.00	3,572.90	3,373.60	3,505.80
Total bases	8.9Gb	8.8Gb	8.4Gb	9.4Gb	9.5Gb	9.5Gb	8.1Gb	7.0Gb	14.6Gb

Table S2: RNAseq accessions used for gene prediction of *Colpophyllia natans* and *Siderastrea siderea* genome assemblies.

Run	Average Spot Length	Bases	BioProject	Illumina Platform	Species
SRR14577699	300	5521266300	PRJNA716052	HiSeq 4000	*Colpophyllia natans*
SRR14577700	300	5331261600	PRJNA716052	HiSeq 4000	*Colpophyllia natans*
SRR14577701	300	4657456500	PRJNA716052	HiSeq 4000	*Colpophyllia natans*
SRR14577711	300	5575275900	PRJNA716052	HiSeq 4000	*Colpophyllia natans*
SRR14577722	300	4972934700	PRJNA716052	HiSeq 4000	*Colpophyllia natans*
SRR14577733	300	6110339400	PRJNA716052	HiSeq 4000	*Colpophyllia natans*
SRR14577744	300	4997716500	PRJNA716052	HiSeq 4000	*Colpophyllia natans*
SRR14577755	300	8047522500	PRJNA716052	HiSeq 4000	*Colpophyllia natans*
SRR14577756	300	6621543300	PRJNA716052	HiSeq 4000	*Colpophyllia natans*
SRR14295591	300	6973818600	PRJNA723585	HiSeq X Five	*Colpophyllia natans*
SRR14295592	300	7406209200	PRJNA723585	HiSeq X Five	*Colpophyllia natans*
SRR14295598	300	6548658900	PRJNA723585	HiSeq X Five	*Colpophyllia natans*
SRR14295599	300	7238971800	PRJNA723585	HiSeq X Five	*Colpophyllia natans*
SRR14295602	300	7699264200	PRJNA723585	HiSeq X Five	*Colpophyllia natans*
SRR14577702	300	7414340100	PRJNA716052	HiSeq 4000	*Siderastrea siderea*
SRR14577703	300	6459067200	PRJNA716052	HiSeq 4000	*Siderastrea siderea*
SRR14577704	300	5875051800	PRJNA716052	HiSeq 4000	*Siderastrea siderea*
SRR14577705	300	6464233800	PRJNA716052	HiSeq 4000	*Siderastrea siderea*
SRR14577706	300	7026348900	PRJNA716052	HiSeq 4000	*Siderastrea siderea*
SRR14577707	300	5965790100	PRJNA716052	HiSeq 4000	*Siderastrea siderea*
SRR14577708	300	7806279300	PRJNA716052	HiSeq 4000	*Siderastrea siderea*
SRR14577709	300	7075873500	PRJNA716052	HiSeq 4000	*Siderastrea siderea*
SRR14577710	300	7210988700	PRJNA716052	HiSeq 4000	*Siderastrea siderea*
SRR3111799	200	10937090800	PRJNA307543	HiSeq 2000	*Siderastrea siderea*
SRR3111800	200	11336494000	PRJNA307543	HiSeq 2000	*Siderastrea siderea*
SRR3111801	200	19998296600	PRJNA307543	HiSeq 2000	*Siderastrea siderea*
SRR3111802	200	9996575600	PRJNA307543	HiSeq 2000	*Siderastrea siderea*
SRR3111803	200	12646209800	PRJNA307543	HiSeq 2000	*Siderastrea siderea*
SRR3111804	100	5095161100	PRJNA307543	HiSeq 2000	*Siderastrea siderea*
SRR3111805	200	13831462200	PRJNA307543	HiSeq 2000	*Siderastrea siderea*
SRR3111806	200	9700554200	PRJNA307543	HiSeq 2000	*Siderastrea siderea*
SRR3111807	200	17665039400	PRJNA307543	HiSeq 2000	*Siderastrea siderea*
SRR3111808	200	9629022400	PRJNA307543	HiSeq 2000	*Siderastrea siderea*
SRR3111809	200	15423703200	PRJNA307543	HiSeq 2000	*Siderastrea siderea*
SRR3111810	199	12712562496	PRJNA307543	HiSeq 2000	*Siderastrea siderea*
SRR14295600	300	6767854200	PRJNA723585	HiSeq X Five	*Siderastrea siderea*
SRR14295601	300	6932813100	PRJNA723585	HiSeq X Five	*Siderastrea siderea*

Run	Average Spot Length	Bases	BioProject	Illumina Platform	Species
SRR14295603	300	7289401500	PRJNA723585	HiSeq X Five	*Siderastrea siderea*
SRR14295604	300	6194834400	PRJNA723585	HiSeq X Five	*Siderastrea siderea*
SRR12454619	300	59349812100	PRJNA635110	HiSeq 4000	*Siderastrea siderea*
SRR20761964	300	4066989000	PRJNA865460	HiSeq 2500	*Siderastrea siderea*
SRR20761965	300	3946718700	PRJNA865460	HiSeq 2500	*Siderastrea siderea*
SRR20761966	300	4242785400	PRJNA865460	HiSeq 2500	*Siderastrea siderea*
SRR20761967	300	3280289700	PRJNA865460	HiSeq 2500	*Siderastrea siderea*
SRR20761968	300	2846652900	PRJNA865460	HiSeq 2500	*Siderastrea siderea*
SRR20762002	300	5687082900	PRJNA865460	HiSeq 2500	*Siderastrea siderea*
SRR20762003	300	4190301900	PRJNA865460	HiSeq 2500	*Siderastrea siderea*
SRR20762004	300	3145650300	PRJNA865460	HiSeq 2500	*Siderastrea siderea*
SRR20762005	300	4494754500	PRJNA865460	HiSeq 2500	*Siderastrea siderea*
SRR20762006	300	3929991900	PRJNA865460	HiSeq 2500	*Siderastrea siderea*
SRR20762007	300	2805511500	PRJNA865460	HiSeq 2500	*Siderastrea siderea*
SRR20762008	300	3002441400	PRJNA865460	HiSeq 2500	*Siderastrea siderea*

Table S3: Genome assemblies used for OrthoFinder (Emms and Kelly 2019) and doubletrouble (Almeida-Silva and Peer 2024) comparative analyses.

Species	Reference
Siderastrea siderea	This study
Colpophyllia natans	This study
Dendrogyra cylindrus	This study
Acropora cervicornis	(Locatelli *et al.* 2023)
Acropora loripes	(Salazar *et al.* 2022)
Acropora millepora	(Fuller *et al.* 2020)
Acropora palmata	(Locatelli *et al.* 2023)
Amplexidiscus fenestrafer	(Wang *et al.* 2017)
Astrangia poculata	(Stankiewicz *et al.* 2023)
Catalaphyllia jardinei	(Yu *et al.* 2022)
Desmophyllum pertusum	(Herrera and Cordes 2023)
Discosoma sp.	(Wang *et al.* 2017)
Fungia fungites	(Ying *et al.* 2018)
Galaxea fascicularis	(Ying *et al.* 2018)
Goniastrea aspera	(Ying *et al.* 2018)
Montipora capitata	(Helmkampf *et al.* 2019)
Orbicella faveolata	(Prada *et al.* 2016)
Platygyra daedalea	(Liew *et al.* 2016)
Pocillopora meandrina	(Stephens *et al.* 2022)
Pocillopora verrucosa	(Buitrago-López *et al.* 2020)
Porites compressa	(Stephens *et al.* 2022)
Porites lobata	(Noel *et al.* 2023)
Stylophora pistillata	(Voolstra *et al.* 2017)

Table S4: All duplicate classifications identified by doubletrouble (Almeida-Silva and Peer 2024). The "full" classification schema of doubletrouble was only run in *Colpophyllia natans, Dendrogyra cylindrus*, and *Siderastrea siderea* due to compatibility of input files. SD=Segmental duplicates, TD=Tandem duplicates, PD=Proximal duplicates, TRD=Transposon-derived duplicates, rTRD=Retrotransposon-derived duplicates, dTRD=DNA transposon-derived duplicates, and DD=Dispersed duplicates. Species in bold were assembled and annotated in this study. All included taxa are listed in **Table S3**.

Species	Assembly Size (Mb)	SD	TD	PD	TRD		DD
					rTRD	dTRD	
Dendrogyra cylindrus	**526**	**677**	**7468**	**4884**	**874**	**4385**	**8053**
Colpophyllia natans	**398**	**963**	**4948**	**3338**	**844**	**4378**	**6884**
Siderastrea siderea	**822**	**2447**	**7480**	**8699**	**1061**	**4283**	**17659**
Acropora cervicornis	309	192	3244	2610	0		16858
Acropora millepora	475	1413	4553	3405	4159		10516
Acropora loripes	402	780	3847	2945	4188		8321
Acropora palmata	336	222	7146	2725	3844		10807
Astrangia poculata	458	849	8362	6204	5978		14276
Catalaphyllia jardinei	651	166	4117	2374	6602		13842
Desmophyllum pertusum	557	754	5625	5459	5500		8257
Fungia fungites	606	24	6353	3417	4595		10996
Goniastrea aspera	764	0	4642	2954	4916		12271
Galaxea fascicularis	334	0	2190	803	3795		7802
Montipora capitata	644	1358	4287	6004	5411		27064
Orbicella faveolata	486	0	8694	2492	4324		3947
Porites compressa	528	1103	7055	6137	4967		14076
Platygyra daedalea	843	14	3585	1741	4176		7779
Porites lobata	646	1327	6743	6687	5272		15394
Pocillopora meandrina	349	1867	5653	5415	5027		7147
Pocillopora verrucosa	381	24	6224	2652	5281		6852
Stylophora pistillata	398	19	4345	2001	5011		6445

Supplemental Figures

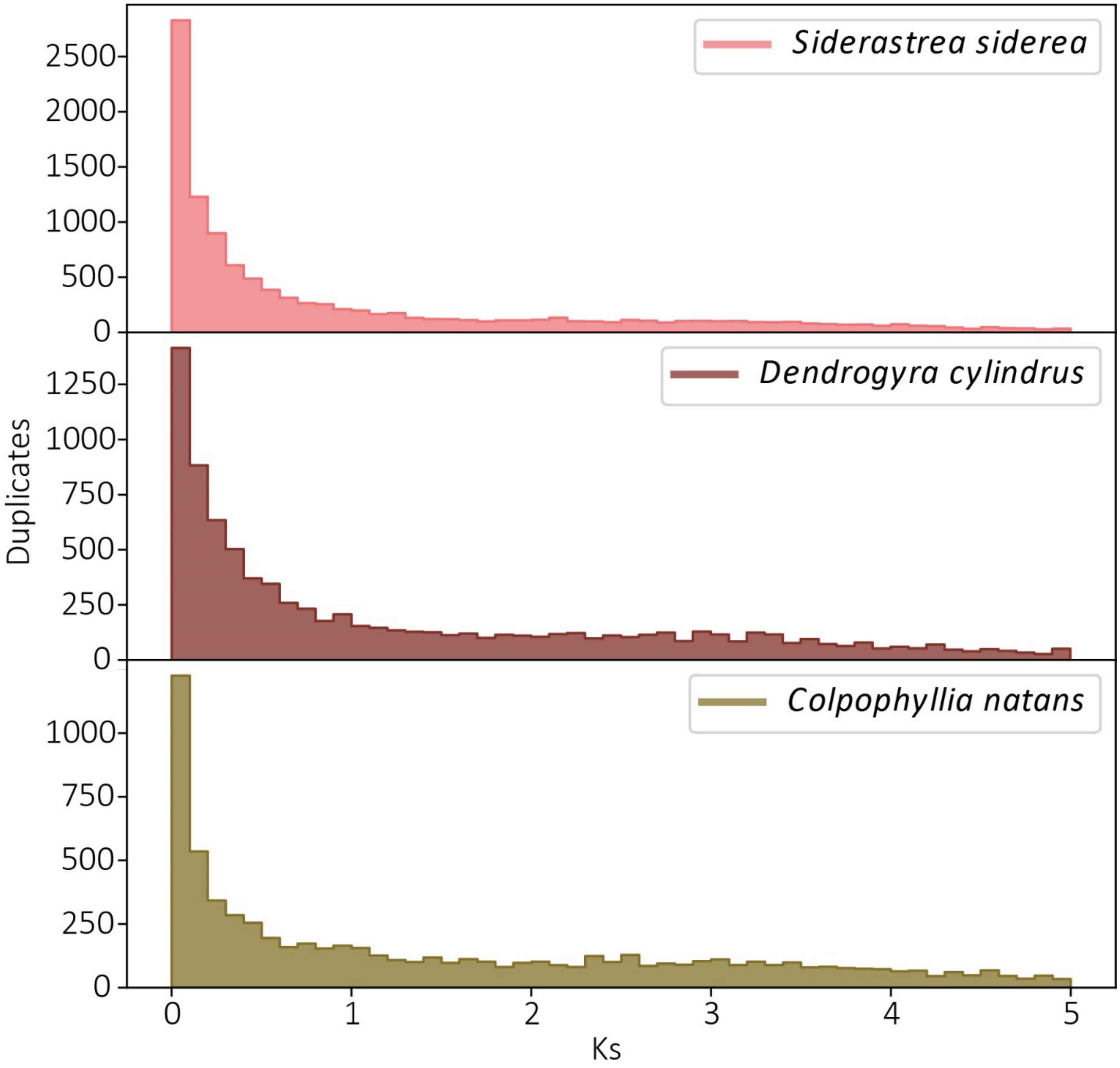

Fig. S1: Ks distribution plots generated by the wgd pipeline (Zwaenepoel and Van De Peer 2019). Ks plots were generated using the longest CDS transcript for each gene in each species. A secondary hump in the Ks distributions would support the presence of a whole genome duplication event. None of the three species here possess distributions that characterize whole genome duplications.

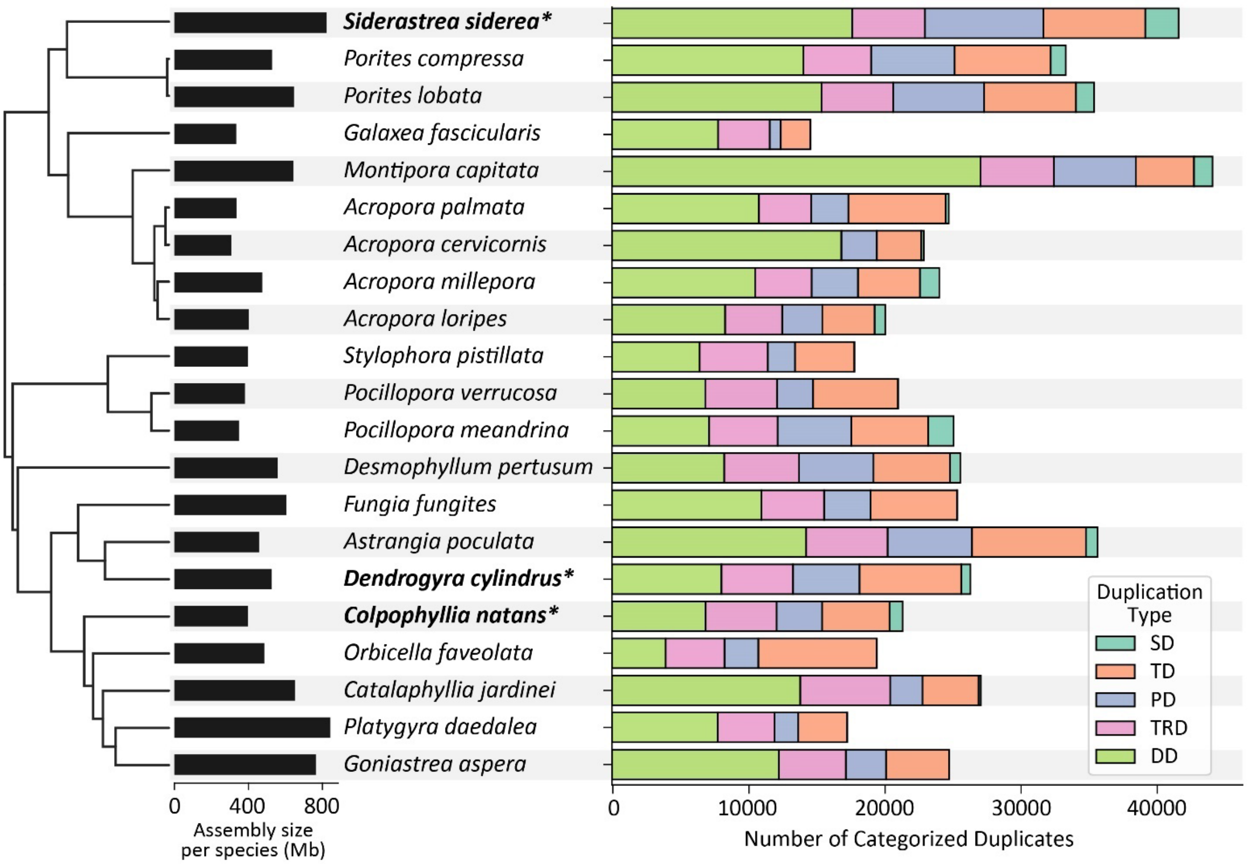

9 *Genomes assembled in present study

Fig. S2: Gene duplication classes as identified by doubletrouble. Gene duplications were assigned duplication classes by doubletrouble (Almeida-Silva and Peer 2024). Gene duplication is closely related to genome size, which is depicted to the left of species names. SD=Segmental duplication, TD=Tandem duplication, PD=Proximal duplication, TRD=Transposon-derived duplication, and DD=Dispersed duplication. The focal taxa assembled in the present study are indicated by bold font and asterisks (*). All included taxa are listed in **Table S3**.

10

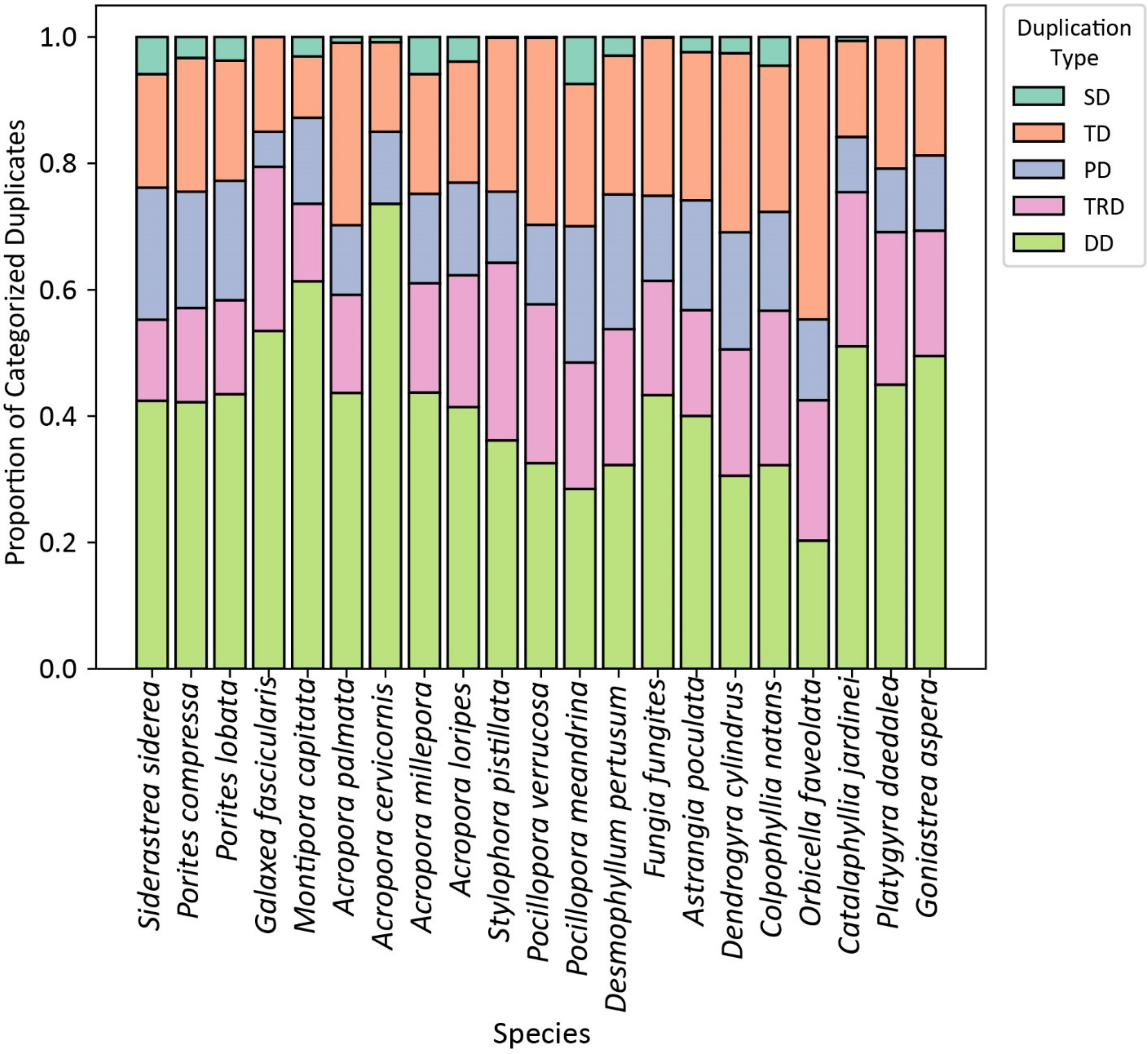

Fig. S3: The proportion of paralogs assigned to each duplication category by doubletrouble (Almeida-Silva and Peer 2024). SD=Segmental duplication, TD=Tandem duplication, PD=Proximal duplication, TRD=Transposon-related duplication, DD=Dispersed duplication. All included taxa are listed in **Table S3**.
